# The Significance of Endometrial Scratching for Clinical Pregnancy Rate in Long Agonist and Antagonist Protocols

**DOI:** 10.3390/medicina55090527

**Published:** 2019-08-23

**Authors:** Rimantas Gricius, Greta Balciuniene, Lina Jakubauskiene, Diana Ramasauskaite

**Affiliations:** 1 JSC Baltic Amrican Clinic, 10103 Vilnius, Lithuania; 2 Vilnius University Hospital Santaros Klinikos, 08661 Vilnius, Lithuania; 3 Clinic of Obstetrics, and Gynecology, Institute of Clinical Medicine, Faculty of Medicine, Vilnius University, 03101 Vilnius, Lithuania

**Keywords:** endometrial scratching, local endometrial injury, in vitro fertilization

## Abstract

*Background and Objectives*: Assisted reproductive techniques today have helped many infertile couples achieve their desired pregnancy. However, unsuccessful implantation is usually the key failure in in vitro fertilization cycles. Many factors are now being studied to improve the implantation rate, one being endometrial artificial injury (endometrial scratching). The purpose of this study was to assess whether local endometrial artificial injury improved pregnancy rate, in long agonist and antagonist protocols. *Materials and Methods*: A retrospective analysis was carried out at the JSC Baltic American Clinic from January 1, 2016 to December 31, 2018. Women who were undergoing in vitro fertilization treatment were enrolled in the study. Medical data including demographic factors, menstrual history, and anamnesis of infertility were collected from medical records. Subjects were divided into intervention and control groups. The outcome of this study was clinical pregnancy rate (CPR). *Results*: A total of 137 women presenting with primary or secondary infertility were enrolled in the study. Clinical pregnancy was observed in 44/71 (61.9%) patients in the intervention group and 33/66 (50%) in the group without endometrial scratching (*p* value = 0.16). CPR in the intervention group was significantly higher in the patient group that had undergone ovarian stimulation with a long agonist protocol rather than the antagonist protocol (73.8% vs. 41.4%; *p* value = 0.006). In the multivariable logistic regression model, previously failed in vitro fertilization (IVF) (odds ratio (OR) = 0.07, 95% confidence interval (CI) 0.01–0.47), stimulation with the long agonist protocol (OR = 19.89, 95% CI 3.57–18.63), antagonist protocol (OR = 0.05, 95% CI 0.01–0.34), number of embryos (OR = 1.37, 95% CI 1.05–1.38), and number of blastocysts (OR = 2.96, 95% CI 1.43–8.36) remained important independent prognostic factors for clinical pregnancy. *Conclusions*: Our study findings indicate that randomized controlled studies are required to define patients for this procedure and to prove the effect and safety of local endometrial injury, before it is introduced to routine clinical practice.

## 1. Introduction

With regard to improved assisted reproductive techniques, many infertile couples nowadays have better opportunities to achieve desired pregnancy. However, repeated in vitro fertilization (IVF) treatment failure is a result of unsuccessful implantation. The development of high-quality embryos and adequate endometrial receptivity are the main limiting factors to successful implantation [1], because even high-grade embryos often fail to implant, resulting in an implantation rate of approximately 25–30% per embryo transfer [2,3]. Many researchers are working to find factors that would improve the implantation rate. Some evidence has shown the superiority of local endometrial injury (LEI) and blastocyst transfer in cases when a couple experiences recurrent IVF failure [4].

Local endometrial injury can improve implantation in three basic ways. First, endometrial scratching modulates the expression of a variety of genes that are involved in the preparation of the endometrium for embryo implantation. Some of these genes are *MUC1*, *crystallin αB*, *APOD*, and *PLA2* [5]. Additionally, endometrial scratching causes a local inflammatory response, which increases the production of proinflammatory cytokines including tumor necrosis factor-α, an endometrial chemokine, macrophage inflammatory protein-1β, and osteopontin. These cytokines have a huge impact on the success of implantation [6]. Moreover, endometrial injury promotes decidualization and improves endometrial receptivity, which leads to better interactions between the endometrium and the embryo [7,8].

The present study was undertaken to assess whether local endometrial artificial injury improved pregnancy rate in long agonist and antagonist protocols and to analyze important prognostic factors for clinical pregnancy.

## 2. Materials and Methods

A retrospective analysis was carried out at the JSC Baltic American Clinic from January 1, 2016 to December 31, 2018. Women who signed informed consent and had undergone the in vitro fertilization procedure were enrolled in this study. All patients were treated in the same clinic and all procedures were performed by the same gynecologist and embryologist. Participants were not included in the study if there was data missing or if informed consent was not signed.

Ethical clearance was obtained from local Institutional Ethics Committee of JSC Baltic American Medical and Surgical Clinic (Approval Number 15/12, approved on 18 December 2015) and written informed consent was obtained from all participants.

Medical data were collected from JSC Baltic American Clinic by a retrospective review of medical records including age, body mass index (BMI), menarche, menstrual cycle length, type and duration of infertility, previous IVF and intrauterine insemination (IUS), sperm parameters, stimulation protocol, the number of oocytes retrieved, the quantity and quality of the embryos and the number of blastocysts. Follicle stimulating hormone (FSH) on day 3 of the menstrual cycle was used to assess ovarian reserve. According to published research, endometrial scratching was offered in routine clinical practice until 2018. Based on patient agreement to perform endometrial scratching, participants were divided into two groups. Women underwent ovarian stimulation according to the long mid-luteal phase GnRH agonist protocol or the GnRH antagonist protocol. The doses of gonadotropins were adjusted according to ovarian response. The ovarian response was monitored by serial transvaginal ultrasound examinations. When the leading follicle was ≥18 mm in diameter or two follicles were larger than 16 mm, recombinant chorionic gonadotropin (rhCG) was injected subcutaneously. Oocyte retrieval was performed 36 hours after rhCG injection. Two cleavage stage best available embryos were transferred on the second day after ovarian puncture. Micronized progesterone was administered vaginally in a dose of 200 micrograms three times daily for luteal phase support. Serum human chorionic gonadotropin-β was measured 14 days after oocyte retrieval and pregnancy was confirmed through transvaginal ultrasound at 5–6 weeks.

Local endometrial scratching was performed using an endometrial sample catheter in the mid luteal phase about seven days prior to menstruation. It was passed through the cervix up to the uterine fundus and moved back and forth between the fundus and internal orifice in the front and back walls of the uterus as well as in the left and right sides, at least three to four times.

The outcome of this study was the clinical pregnancy rate (CPR), which was proven using vaginal ultrasound when viable intrauterine pregnancy was seen.

Statistical analysis was performed using R version 3.5.3 (R Foundation for Statistical Computing, Vienna, Austria). The Shapiro–Wilk test was used to assess the normality of the arithmetic data distributions. The demographic and clinical characteristics were compared through the nonparametric Mann–Whitney U test for continuous variables and presented as median values (range). Categorical variables were compared using a Fisher’s exact test and presented as numbers (%). Associations were tested by the Chi-square test for categorical variables, the *t*-test for continuous variables that were normally distributed, and the Mann–Whitney U test for nonparametric continuous variables. Univariate and multivariable logistic regressions were performed to determine the independent relationships of the patient characteristics and CPR. All *p* values <0.05 were considered significant.

## 3. Results

A total of 137 women presenting with primary or secondary infertility were enrolled in the study, of which 71 patients (51.8%) experienced endometrial scratching. The participating women were compared according to age, body mass index, menstrual history, and other baseline characteristics of infertility (Table 1 and Table 2).

Clinical pregnancy was observed in 44 (61.9%) patients in the intervention group and 33 (50%) in the group without endometrial scratching. The clinical pregnancy rate was higher in the intervention group, but the difference was not significant (*p* value = 0.16). We evaluated which stimulation protocol could be considered as the most effective with endometrial scratching (Table 3). While analyzing the influence of the stimulation protocol only in the intervention group, we found that ovarian stimulation with the long agonist protocol was superior to the antagonist protocol in increasing CPR (73.8% vs. 41.4%, *p* value = 0.006). In addition, we could see a tendency that women in the long agonist protocol group became pregnant more frequently in cases when endometrial scratching was performed (73.8% vs. 54.3%; *p* value = 0.07).

Analyzing the intervention group, we did not observe a statistically significant difference between the cases where women had scratching with the first IVF cycle or after failed IUI cycles. In contrast, women with previously failed IVF showed worse conceiving results than women who had undergone scratching with the first IVF cycle (47.1% vs. 73%, *p* value = 0.03). Furthermore, no statistically significant differences were noticed between the conception and non-conception cycles in the women’s BMI, menarche, duration of infertility, causes of infertility, and total gonadotrophin dose. There were statistically significant differences between the groups in terms of the women’s age, number of oocytes retrieved, number of embryos, number of good quality embryos, and number of blastocysts (Table 4).

Previously failed IVF, women’s age, type of stimulation protocol, number of oocytes retrieved, number of embryos, number of good-quality embryos, and number of blastocysts were found to be associated with clinical pregnancy using univariate logistic regression. In the multivariable logistic regression model, only previously failed IVF, type of stimulation protocol, number of embryos, and number of blastocysts remained as important independent prognostic factors for clinical pregnancy (Table 5).

Multivariable regression analysis including the aforementioned significant variables at the same time was performed. By using this approach, all of the included variables remained independent prognostic factors for clinical pregnancy.

## 4. Discussion

Local endometrial injury is an intervention which is broadly discussed in the literature and could improve the pregnancy rates for women undergoing IVF due to infertility. LEI is presented in the literature as a cost-effective, cheap, and well-tolerated procedure that can be used in patients undergoing IUI/IVF to increase the probability of pregnancy and live birth rate [9].

Multiple studies have been performed to explore the LEI effect on conceiving, but the scratching method, population factors, and heterogeneity in design show that it still remains unclear as to whether endometrial injury should be used [10,11]. However, this method is advised in New Zealand, Australia, and the UK by 83% of clinicians working in fertility clinics [12].

The role of endometrial injury was first studied in a prospective study by Barash et al., where the authors concluded that women with repeated endometrial biopsies were twice as likely to conceive when compared to the control group [13]. This study was followed by many other studies focused on the mechanism and benefit of endometrial local injury. Most of these studies were criticized because of their small sample, methodological flaw and lack of randomization. A first large multicenter randomized controlled trial was published in January 2019 [14]. This study demonstrated no benefit of endometrial scratching in an unselected infertile population undergoing IVF. Thus, it is still unclear as to which group of patients could be candidates for LEI. Some authors have suggested endometrial injury in cases with failed IUI or IVF cycles. Mahran et al. proposed LEI for women with the first IVF cycle, because in their study, LEI improved implantation up to 22.4% when compared to 18.7% in the control group [15]. In contrast, another study performed by Gibreel et al. showed the superiority of LEI only after two or more failed IVF cycles (odds ratio: 3.74, 95% CI: 1.49–7.39) [16]. Meta-analysis of seven controlled studies with 2062 participants showed that endometrial biopsy or scratch increased the CPR more than twice, especially in patients with recurrent IVF failure [17]. Contrary to this meta-analysis, a recent systematic review declared that the results of these studies failed to fully support the role of endometrial scratching as an effective treatment for women with recurrent implantation failures [11].

Women with failed IUI cycles were recruited for LEI as it increased CPR in the endometrial scratching group when compared to the controls (25.5% vs. 14.1%, *p* value 0.03) [18]. A meta-analysis of eight randomized controlled trials showed that endometrial scratching performed during the follicular phase of the same cycle of IUI was associated with a higher clinical pregnancy (OR 2.27) and ongoing pregnancy rates (OR 2.04) [19]. In our study, we examined the influence of failed previous IVF, but did not observe the influence of previous IUI on CPR.

To the best of our knowledge, this is the first study to have investigated the impact of ovarian stimulation protocol on CPR when endometrial scratching is performed. Long agonist and antagonist protocols affect hormonal changes, synchronization of follicular recruitment, growth and oocyte maturation differently. We think that endometrial local injury could modulate different endometrial gene expression in each protocol. In our study, we found that ovarian stimulation with a long agonist protocol was superior to the antagonist protocol in increasing CPR in women who had undergone endometrial scratching.

An important limitation of our trial is that we used a small sample of the study population, which limited us in achieving statistically significant results of some measurements. Relatively small sample size was determined by the selection of the participants who had undergone the procedures by the same gynecologist and embryologist in a single institution. Although the retrospective study design seems to be another potential limitation, the described selection of participants represents a homogenous group of patients.

## 5. Conclusions

Our study findings, along with many other controversial studies, indicate that more randomized controlled studies are required to define patients for this procedure, as well as prove the effect and safety of LEI before it is introduced into routine clinical practice.

## Figures and Tables

**Table 1:** The demographic and clinical characteristics of patients.

Patients’ Characteristics	Scratched (*n* = 71)	Non-Scratched (*n* = 66)	*p* Value
Age (average ± SD) (years)	34.61 ± 5.06	34.77 ± 4.4	0.84
BMI (average ± SD) (kg/m^2^)	22.22 ± 3.69	22.01 ± 3.56	0.73
***Menstrual history***			
Menarche	13.63 ± 1.28	13.55 ± 1.2	0.68
Regular menstrual cycle (*n*, %)	68 (95.8)	62 (93.9)	0.63
Irregular menstrual cycle (*n*, %)	3 (4.2)	4 (6.1)	0.63
***Anamnesis of infertility***			
Primary (*n*, %)	51 (71.8)	44 (66.7)	0.51
Secondary (*n*, %)	20 (28.2)	21 (33.3)	0.51
Duration of infertility (years)	5.65 ± 4.55	4.86 ± 3.6	0.26
Male factor infertility (*n*, %)	32 (45.1)	32 (48.5)	0.69
FSH (IU/L)	8.95 ± 4.8	8.6 ± 3.62	0.78
Previously failed IVF cycles (*n*, %)	34 (47.9)	31 (48.5)	0.94
Previously failed IUI (*n*, %)	20 (28.2)	27 (40.9)	0.12
Total gonadotrophin dose	2254.93 ± 493.88	2136.17 ± 709.79	0.26
Long agonist protocol (*n*, %)	42 (59.2)	36 (54.5)	0.59
Antagonist protocol (*n*, %)	29 (40.8)	30 (45.5)	0.59
Number of oocytes retrieved	11.45 ± 8.12	12.15 ± 7.89	0.61
Number of embryos	7.94 ± 6.71	7.56 ± 4.84	0.7
Number of good-quality embryos	2.11 ± 2.87	1.52 ± 2.05	0.16
Number of blastocysts	7.52 ± 6.33	6.91 ± 4.31	0.51

The *p* value showed no significant differences between the two groups. SD, standard deviation; *n*, numbers; BMI, body mass index; IVF, in vitro fertilization; IUI, intrauterine insemination.

**Table 2:** Differences in the characteristics between patients who had undergone long agonist and antagonist protocols.

Patients’ Characteristics	Long Agonist Protocol (*n* = 78)	Antagonist Protocol (*n* = 59)	*p* Value
Age (average ± SD) (years)	33.99 ± 4.2	35.61 ± 5.26	0.06
BMI (average ± SD) (kg/m^2^)	21.61 ± 3.47	22.8 ± 3.74	0.06
***Menstrual history***			
Menarche	13.6 ± 1.26	13.57 ± 1.22	0.9
Regular menstrual cycle (*N*, %)	74 (94.87)	56 (94.92)	0.99
Irregular menstrual cycle (*N*, %)	4 (5.13)	3 (5,08)	0.99
***Anamnesis of infertility***			
Primary (*n*, %)	54 (62.23)	41 (69.49)	0.94
Secondary (*n*, %)	23 (37.38)	18 (30.51)	0.94
Duration of infertility (years)	5.23 ± 3.46	5.32 ± 4.9	0.9
FSH (IU/L)	8.93 ± 4.94	8.63 ± 3.52	0.81
Male factor infertility (*n*, %)	36 (46.15)	28 (47.46)	0.88
Previously failed IVF (*n*, %)	38 (48.72)	28 (47.46)	0.88
Previously failed IUI (*n*, %)	26 (33.33)	21 (35.59)	0.78
Number of oocytes retrieved	12.38 ± 7.85	11.0 ± 8.17	0.32
Number of embryos	7.97 ± 5.89	7.47 ± 5.87	0.62
Number of good-quality embryos	1.97 ± 2.72	1.62 ± 2.22	0.41
Number of blastocysts	7.49 ± 5.66	6.88 ± 5.17	0.51

The *p* value showed no significant differences between the groups. SD, standard deviation; *n*, numbers; BMI, body mass index; IVF, in vitro fertilization; IUI, intrauterine insemination.

**Table 3:** Clinical pregnancy rate (CPR) depending on the stimulation protocol.

Stimulation Protocol	CPR (%)		*p* Value
	Scratched	Non-Scratched	
Long agonist protocol	31/42 (73.8%)	19/35 (54.3%)	0.07
Antagonist protocol	12/29 (41.4%)	15/30 (50%)	0.51
*p* value	0.006	0.73	

**Table 4:** Differences in the patient characteristics between conception in vitro fertilization (IVF) cycles and non-conception IVF cycles.

Patient Characteristics	Conception IVF Cycles (*N* = 43)	Non-Conception IVF cycles (*N* = 28)	*p* Value
Previously failed IVF (*n*, %)	16 (37.2)	18 (64.3)	0.03
Previously failed IUI (*n*, %)	13 (30.2)	7 (25)	0.63
Age (average ± SD) (years)	32.77 ± 4.01	37.43 ± 5.25	0.0002
BMI (average ± SD) (kg/m^2^)	21.61 ± 4.03	23.17 ± 2.92	0.06
Menarche	13.51 ± 1.33	13.82 ± 1.19	0.31
Duration of infertility (years)	5.21 ± 3.37	6.32 ± 5.94	0.37
FSH (IU/L)	8.19 ± 3.55	9.49 ± 4.87	0.31
Male factor infertility (*n*, %)	18 (51.9)	14 (50)	0.5
Total gonadotrophin dose	2192.44 ± 446.03	2350.89 ± 554.21	0.21
Number of oocytes retrieved	13.30 ± 7.88	8.61 ± 7.79	0.02
Number of embryos	9.51 ± 6.53	5.54 ± 6.36	0.01
Number of good-quality embryos	2.79 ± 2.99	1.07 ± 2.34	0.009
Number of blastocysts	9.19 ± 6.47	4.97 ± 5.25	0.003

*n*, numbers; BMI, body mass index; IVF, in vitro fertilization; IUI, intrauterine insemination.

**Table 5:** Univariate and multivariable logistic regression analysis assessing the predictors of clinical pregnancy after IVF in women with endometrial scratching.

Independent Variable	Univariate Regression		Multivariable Regression	
	Odds Ratio (95% CI)	*p* Value	Odds Ratio (95% CI)	*p* Value
Previously failed IVF	0.33 (0.12–0.87)	0.03	0.07 (0.01–0.47)	0.01
Previously failed IUI	1.3 (0.45–3.97)	0.63	3.65 (0.57–3.42)	0.2
Age	0.81 (0.71–0.91)	0.0005	0.84 (0.67–1.01)	0.08
BMI	0.88 (0.76–1.02)	0.09	0.84 (0.66–1.06)	0.16
Menarche	0.82 (0.56–1.2)	0.32	0.72 (0.47–1.58)	0.36
Duration of infertility	0.95 (0.85–1.05)	0.32	1.01 (0.82–1.23)	0.92
Total gonadotrophin dose	0.99 (0.99–1)	0.19	0.99 (0.99–1.01)	0.16
Long agonist protocol	3.99 (1.48–11.3)	0.007	19.89 (3.57–18.63)	0.002
Antagonist protocol	0.25 (0.09–0.69)	0.007	0.05 (0.01–0.34)	0.002
Number of oocytes retrieved	1.09 (1.01–1.18)	0.02	1.01 (0.89–1.02)	0.09
Number of embryos	1.14 (1.02–1.27)	0.02	1.37 (1.05–1.38)	0.05
Number of good-quality embryos	1.43 (1.07–1.91)	0.02	1.47 (0.89–2.58)	0.15
Number of blastocysts	1.18 (1.04–1.34)	0.01	2.96 (1.43–8.36)	0.01

CI, confidence interval; BMI, body mass index; IVF, in vitro fertilization; IUI, intrauterine insemination.
